# After Fish Eat Cheese

**Published:** 1898-06

**Authors:** B. Fincke

**Affiliations:** Brooklyn


### AFTER FISH EAT CHEESE.

B. Fincke, M. D., Brooklyn.

Once upon a time there lived in a small town in Germany a good old doctor who was called up in the night. He opened the window, and the servant of the gracious lady living in the castle on the hill shouted up to him that he was wanted immediately. But the doctor, frequently called for nothing particular, shouted down: "What has she eaten?"

"Fish!" was the answer. "Tell her to eat cheese!"* shouted the doctor down again, closed the window and went to bed. Next morning the servant came to tell the doctor that the lady had died that night. "Has she eaten cheese?" asked the doctor. "No," said the servant. "The gracious lady was greatly indignant at your language, and then she died." Now in the German language only men do eat, but not animals; they are said to "fressen," *i. e.*, eat greedily. The fact was the doctor was indignant at the old lady for overeating herself with fish, and used the rude expression in German, "Sie soll Käs fresse," which cost the lady's life.

From this veritable story the moral is to be drawn:

1. Do not eat too much fish at once.

2. If you do, eat cheese to help its digestion.

3. The ptomaines are not in the fish, but in the heads of ignorant doctors.

